# 

**Published:** 1888-02

**Authors:** 


### HALL'S
### JOURNAL OF HEALTH

TRUTH DEMANDS NO SACRIFICE; ERROR CAN MAKE NONE.

VOL. 35. FEBRUARY, 1888. No 2.

### SPECIAL.

Owing to the unexpected absence in Europe of the Editors of *Earnest Words*, it has been deemed advisable to suspend its further publication for the present. In order, however, that those whose paid period of subscription has not expired, shall suffer no disappointment by this suspension, an arrangement has been made with HALL'S JOURNAL OF HEALTH to fill their several terms with issues of this journal, and we trust that this arrangement will prove satisfactory to all parties concerned.

### BOOK NOTICES.

HEALTH AND HOME.—Health and Home Publishing Co., Chicago, Ill. Quarterly, $1 per year. This new quarterly appeals to public favor in elegant attire and a most excellent table of contents. Its leading article, "The Education of the Senses," shows that this publication recognizes in man the existence of those soul faculties which belong to the spiritual side of his nature, and endure through all changes of time and circumstance. Its Home Department is very full and well chosen. We commend *Health and Home* to the intelligent and thoughtful.

The following excellent monthlies have been laid upon our table :

"WOMAN."—Woman Publishing Co., New York City, $2.75 a year.

JOURNAL OF MAN.—6 James St., Boston, $1.00 per year.

ANNALS OF GYNECOLOGY.—Rockwell & Churchill, Boston, $2 per annum.

THE COSMOPOLITAN.—Schlich & Field Co., New York, $2 per annum.

HORTICULTURAL ART JOURNAL.—Stecher Lithographic Co., 336 St. Paul St., Rochester, N. Y., $3 per annum.

VICK'S ILLUSTRATED MONTHLY.—James Vick, Seedsman, Rochester, N. Y., $1.25 per annum.

TABLE TALK.—Table Talk Publishing Co., Philadelphia, $1 per annum.

THE CENTURY.—Circulation has entirely fallen off, this year, at our office, but we suppose it still circulates in some remote localities.

We are in receipt of the following treatises, etc.:

MORALS & ART, by Anthony Comstock.

THE MOVEMENT CURE, by Mark S. Purdy, M. D., Corning, N. Y.

VICK'S FLORAL GUIDE, 1888.—None better. Rochester, N. Y.

OXYGEN AS A THERAPEUTIC AGENT, by P. D. Rothwell, M. D., Denver, Col.

STATISTICAL REPORT OF 5.700 CASES OF EAR DISEASES, ETC., ETC., by S. B. Bishop, M. D., Chicago.

PROGRESSIVE MUSCULAR ATROPHY, by J. B. Marvin, M. D., Louisville, Ky.

THE INTERNATIONAL MEDICAL AND SURGICAL SYNOPSIS, St. Louis, Mo.

